# Multiple breast cancer risk variants are associated with differential transcript isoform expression in tumors

**DOI:** 10.1093/hmg/ddv432

**Published:** 2015-10-15

**Authors:** Jennifer L. Caswell, Roman Camarda, Alicia Y. Zhou, Scott Huntsman, Donglei Hu, Steven E. Brenner, Noah Zaitlen, Andrei Goga, Elad Ziv

**Affiliations:** 1 Department of Medicine,; 2 Institute for Human Genetics,; 3 Helen Diller Family Comprehensive Cancer Centerand,; 4 Department of Cell and Tissue Biology, University of California, San Francisco, CA, USA,; 5 Department of Medicine, Division of Medical Oncology, Stanford University, Stanford, CA, USA and; 6 Department of Plant and Microbial Biology, University of California, Berkeley, CA, USA

## Abstract

Genome-wide association studies have identified over 70 single-nucleotide polymorphisms (SNPs) associated with breast cancer. A subset of these SNPs are associated with quantitative expression of nearby genes, but the functional effects of the majority remain unknown. We hypothesized that some risk SNPs may regulate alternative splicing. Using RNA-sequencing data from breast tumors and germline genotypes from The Cancer Genome Atlas, we tested the association between each risk SNP genotype and exon-, exon–exon junction- or transcript-specific expression of nearby genes. Six SNPs were associated with differential transcript expression of seven nearby genes at FDR < 0.05 (*BABAM1*, *DCLRE1B/PHTF1*, *PEX14*, *RAD51L1, SRGAP2D* and *STXBP4*). We next developed a Bayesian approach to evaluate, for each SNP, the overlap between the signal of association with breast cancer and the signal of association with alternative splicing. At one locus (*SRGAP2D*), this method eliminated the possibility that the breast cancer risk and the alternate splicing event were due to the same causal SNP. Lastly, at two loci, we identified the likely causal SNP for the alternative splicing event, and at one, functionally validated the effect of that SNP on alternative splicing using a minigene reporter assay. Our results suggest that the regulation of differential transcript isoform expression is the functional mechanism of some breast cancer risk SNPs and that we can use these associations to identify causal SNPs, target genes and the specific transcripts that may mediate breast cancer risk.

## Introduction

Genome-wide association studies (GWASs) have identified thousands of disease risk-associated single-nucleotide polymorphisms (raSNPs), including, to date, 75 that are associated with breast cancer risk (1). The vast majority of raSNPs are located in noncoding regions of the genome; therefore, they, or SNPs in linkage disequilibrium (LD) with them, are likely to influence risk by affecting the regulation of nearby genes or noncoding RNAs (2,3). To determine their function, investigators have tested their association with expression levels of nearby genes (expression quantitative trait loci, or eQTLs) in *cis* (4–7) or in *trans* (4) and assessed whether SNPs in LD with the index raSNP demonstrate evidence for transcription factor binding or histone methylation (6,8). These methods have uncovered eight eQTL associations (4,7), three associations with the targets of a nearby transcription factor (4) and an enrichment of FOXA1 and ESR1 enhancer-binding sites within the raSNP loci (8).

Another, yet unexplored, mechanism by which raSNPs may affect regulation of nearby genes is through post-transcriptional regulation, such as alternative splicing. Previous work has used genome and transcriptome data from lymphoblastoid cell lines to systematically search for germline variants associated with the expression level of a specific transcript isoform of a gene (9–11). These genome-wide analyses have identified hundreds of splicing quantitative trait loci (splicing QTLs), typically exonic or intronic variants that affect exon skipping, alternative splice site inclusion, or the gene's 5′ or 3′ end sequence (9–11). GWAS variants are modestly enriched for splicing QTLs as well as for eQTLs (9), suggesting that some raSNPs may affect risk by affecting differential transcript expression.

Modification of alternative splicing is known to be important in cancer development (12) and the epithelial-mesenchymal transition (13), and recent work has shown that somatic mutations affecting splicing can act as driver mutations in tumors (14). However, no systematic analysis has examined germline variants affecting cancer risk to identify, which may affect alternative splicing. In this paper, we develop methods to query whether a specific raSNP functions as a splicing QTL of a nearby gene. Using publicly available data from The Cancer Genome Atlas (TCGA) (15), we perform a focused analysis of breast cancer raSNPs, discovering five risk loci that may mediate risk by affecting differential transcript isoform expression.

## Results

### Splicing QTL analysis of breast cancer raSNPs

**Figure 1.**
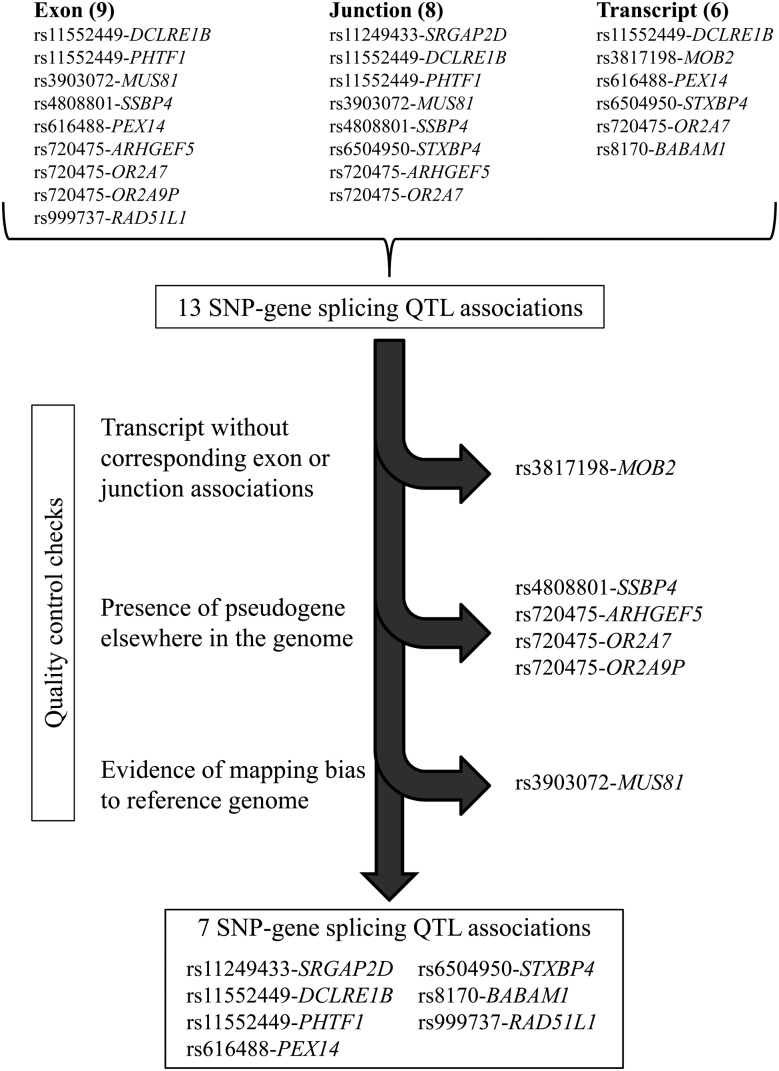
Flowchart for determining splicing QTL associations. We identified 13 SNP-gene associations through exon, junction and whole-transcript association tests with risk-associated SNPs; several associations were identified by multiple methods. After excluding SNP-gene associations that could not be corroborated with other tests, that could be related to the presence of pseudogenes or paralogs or that could have derived from mapping bias to the reference genome, seven SNP-gene associations remained.

We used the RNA-sequencing (RNA-seq) data and matched germline genotypes for 358 estrogen receptor (ER)-positive breast tumors and 109 ER-negative breast tumors from TCGA. For each of the breast cancer raSNPs, we searched for differential transcript isoform expression of nearby genes (Supplementary Material, Table S1), adjusting for overall gene expression, global expression variability (16,17) and genetic ancestry. We used three complementary approaches, testing the association between raSNPs and (1) rank-normalized reads per kilobase per million mapped reads (RPKM) mapping to each exon, (2) rank-normalized reads per million mapped reads (RPM) mapping to each exon–exon junction and (3) rank-normalized expression estimates of reconstructed transcripts of each annotated isoform, as generated by the RSEM algorithm using UCSC transcripts (chosen as its output is available through TCGA) (3) (Supplementary Material, Tables S2–S4). We identified 13 associations with 10 raSNPs using these methods at FDR < 0.05, including 9 exon associations, 8 junction associations and 6 whole-transcript associations; several splicing QTLs were identified by more than one approach (Fig. 1). Q–Q plots showed deviation from normality at the extremes of the *P*-value distributions (Supplementary Material, Fig. S1). When the analysis was repeated in the smaller set of ER-negative tumors, we identified four associations with four raSNPs, including two exon associations, two junction associations and two transcript associations (Supplementary Material, Table S5), all of which were also identified in the ER-positive tumors.

For the exon-specific test, we also tested for differences in raw counts mapping to each exon, using the negative binomial distribution as implemented by the DEXSeq R Bioconductor software package V1.8.0 (18). Of the nine SNP-gene exon associations identified using rank-normalized RPKM values, seven were significant at FDR < 0.05 when using DEXSeq, although the methods identified differing numbers of exons as significant (Supplementary Material, Table S6). One additional exon association (*DCLRE1B*) identified with rank-normalized RPKM values was captured because our test adjusted for overall gene expression with the exon of interest excluded, rather than because of a difference between rank-normalized RPKM values/linear regression versus raw counts/the negative binomial distribution. Given the similarity of the results, we used only normalization and linear regression for the remainder of the analyses.

We next excluded associations that could have resulted from one of three possible sources of error (Fig. 1). Associations identified through whole-transcript reconstruction require a high level of scrutiny because of inherent inaccuracies in transcript assembly, underscored by inconsistency in results from different methods (19). We therefore required associations identified through whole-transcript reconstruction to be supported by significant exon- or junction-specific associations, excluding one association (rs3817198-*MOB2*) that was not consistent with any exon- or junction-specific event at even a nominal level of significance. In contrast, the rs8170-*BABAM1* association, also identified through whole-transcript reconstruction, was supported by increased expression of one exon 1–2 junction (*P* = 1.9 × 10^−4^) and decreased expression of another that used an alternate 3′ acceptor site (*P* = 0.024).

We excluded four associations (two raSNPs) because the gene of interest had a paralog or pseudogene in another part of the genome. If a read can map to two different sections of the genome, the mapping algorithm's inaccuracy in placing it correctly can generate bias exacerbated by genetic variation (20). Rs720475 was identified as a splicing QTL for three genes: *ARHGEF5*, *OR2A7* and *OR2A9P. ARGHEF5* and *OR2A7* are near-identical homologs; in a recent annotation of the genome [Gencode V19 (21)], *OR2A7* has been extended and labeled *ARHGEF34P*. Similarly, *OR2A9P* is included in this region and represented by two pseudogenes 40 kB apart. Thus, the associations between rs720475 and expression of these three genes at least in part reflected difficulties in mapping reads that could come from multiple genes. The associations between rs4808801 and *SSBP4* exons 2–4 were also excluded because of the presence of a retrotransposed pseudogene of *SSBP4* on chromosome 18.

Finally, we excluded one association because of evidence of mapping bias to the reference genome. Mapping algorithms successfully map RNA-seq reads containing the reference allele more frequently than reads containing the alternate allele (22); eQTL and splicing QTL analyses may be susceptible to this bias if the exons contain SNPs in LD with the index raSNP. Four of the splicing QTL loci (including *SSBP4*, already excluded because of the presence of a pseudogene) contained an SNP in LD with the index raSNP (*r^2^* > 0.1) within the associated exon or junction. For each of these loci, we recalculated the association excluding all reads that mapped across such SNPs (Supplementary Material, Table S7). The associations between rs6504950 and *STXBP4* and between rs11552449 and *DCLRE1B* remained significant. However, the associations between rs3903072 and *MUS81* were not significant when excluding the reads that mapped to a pair of SNPs, rs659857 and rs545500. These SNPs are located two base pairs apart in *MUS81* exon 6 and are in perfect LD with each other, an unusual situation that increases the potential for mapping bias.

**Figure 2.**
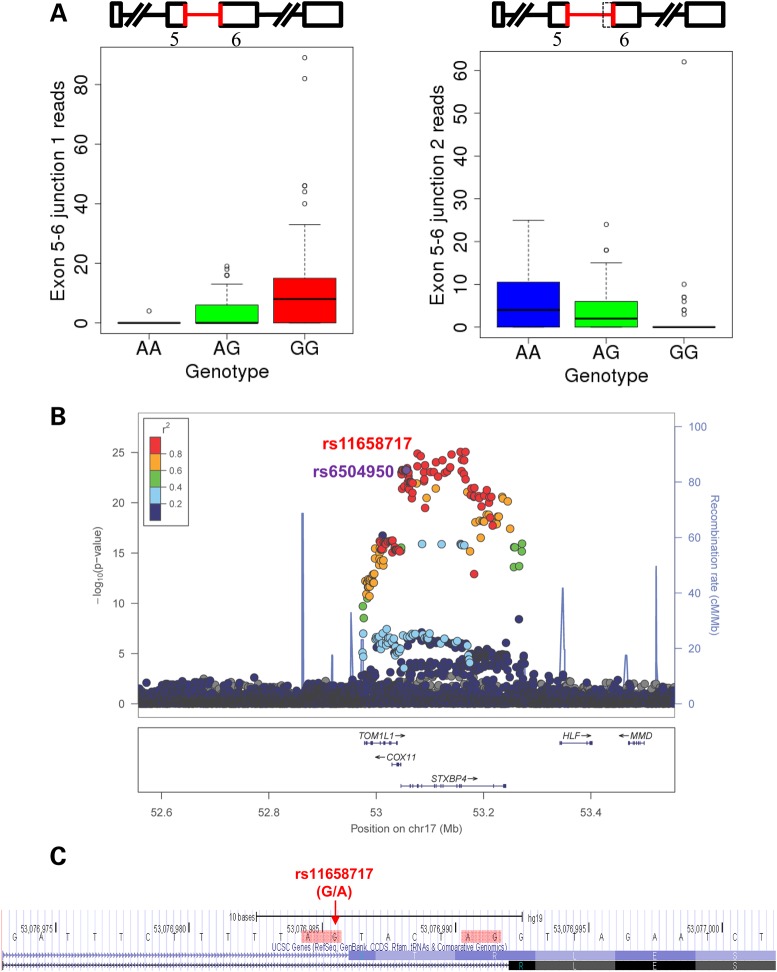
Alternative splice site usage in *STXBP4* exon 6 based on rs6504950 genotype. (**A**) With the rs6504950 risk allele, virtually all *STXBP4* exon 5–6 junction reads map to one junction, whereas with the non-risk allele, virtually all map to the other. (**B**) LocusZoom plot (23) displaying −log_10_ *P*-values for the association of each SNP within the window with *STXBP4* exon 5–6 junction 1 by position. (**C**) The locus of rs11658717, the presumed causal SNP. The two alternative 3′ splice sites are highlighted in red. The minor allele of rs11658717 (G) is in high LD with the risk allele of rs6504950 (A). Screenshot from http://genome.ucsc.edu (24).

**Figure 3.**
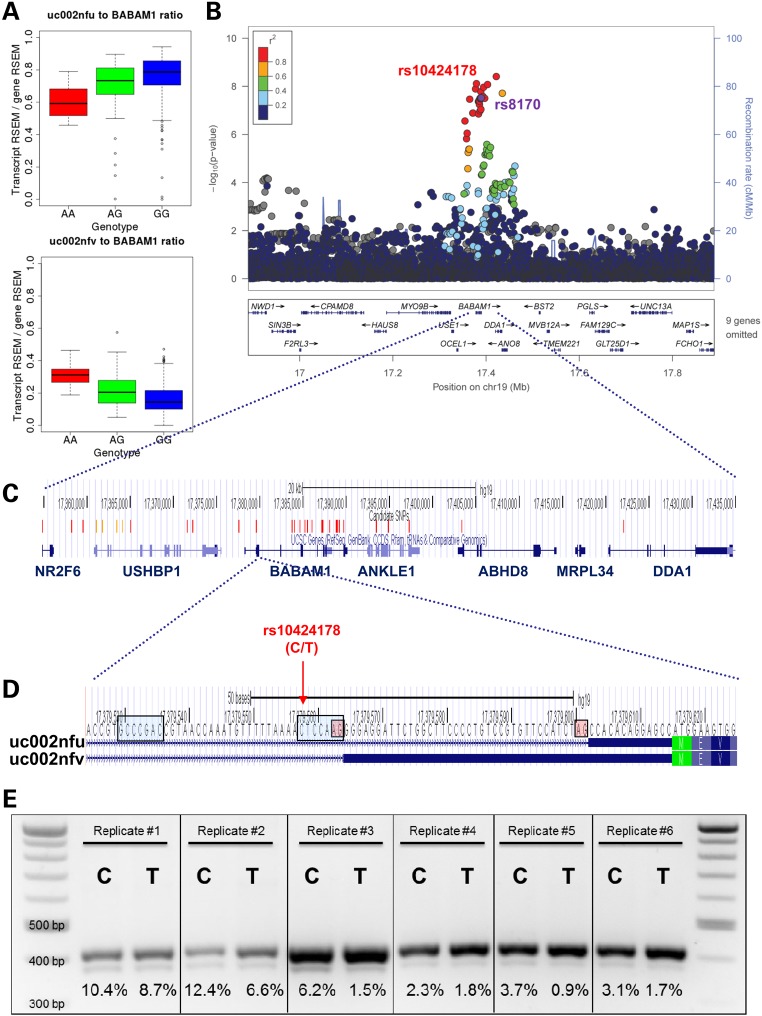
Alternative splice site usage in *BABAM1* exon 2 based on rs8170 genotype. (**A**) Relative expression of RSEM reconstructed *BABAM1* transcript uc002nfu decreases and uc002nfv increases with rs8170 risk genotype. (**B**) LocusZoom plot (23) displaying −log_10_ *P*-values for the association of each SNP within the window with *BABAM1* uc002nfv transcript expression by position. (**C**) Locations of all candidate SNPs, defined as SNPs with *r^2^* > 0.6 with rs8170, or LD unknown but splicing QTL association *P*-value of <1 × 10^−6^. SNPs are colored red if *r^2^* > 0.8 and orange if *r^2^* > 0.6. Screenshot from http://genome.ucsc.edu (24). (**D**) The locus of rs10424178, the presumed causal SNP. The two alternative 3′ splice sites are highlighted in red, and the two alternative branch points as identified by Human Splicing Finder (25) are highlighted in blue. The minor allele of rs10424178 (T) is in high LD with the risk allele of rs8170. Screenshot from http://genome.ucsc.edu (24). (**E**) Results of a six replicates of a minigene reporter vector assay, transfecting the major allele (C) or minor allele (T) of rs10424178. In each well, the lower band represents the shorter *BABAM1* exon 2, as included in transcript uc002nfu, and the upper band represents the longer *BABAM1* exon 2, as included in transcript uc002nfv; the identities of the bands were confirmed by sequencing. The percentages shown below each well are the intensity of the lower band divided by the sum of the intensities of the lower band and the upper band. In all six replicates, the percentage of the total bands represented by the shorter *BABAM1* exon 2 is higher for the major allele than that for the minor (risk) allele.

After excluding the six problematic associations, six raSNPs were associated with exon, junction or whole-transcript expression of seven genes (Table 1). Four of the six loci replicated at *P* < 0.05 in the smaller set of 109 ER-negative tumors from TCGA (Table 1), three at FDR < 0.05. We identified one SNP associated with exon skipping (rs11552449-*DCLRE1B*), two SNPs associated with alternative splice site usage (rs6504950-*STXBP4* and rs8170-*BABAM1*) (Figs 2 and 3), three SNPs associated with more complex exon usage patterns (rs11552449-*PHTF1*, rs616488-*PEX14* and rs999737-*RAD51L1*) and one SNP associated only with an exon–exon junction, which could represent an unannotated alternative splice site or other unannotated pattern of exon usage (rs11249433-*SRGAP2D*).

**Table 1.** Splicing QTLs identified in ER-positive tumors at FDR < 0.05, after exclusions

SNP rsID	Gene	β (ER+)	*P*-value (ER+)	FDR (ER+)	*P*-value (ER−)	Predicted splicing pattern associated with breast cancer risk
	Component					
rs6504950	*STXBP4*					Longer exon 6 (6 bp 5′): ↑ use of 3′ acceptor site at chr17:53 076 993 and ↓ use of chr17:53 076 987
	Exon 5:6 junction 1	−0.73	5.5E − 24	8.3E − 20	1.9E − 11	
	Exon 5:6 junction 2	0.59	1.9E − 23	1.4E − 19	1.6E − 07	
	Transcript uc010dcc	−0.42	3.5E − 11	4.4E − 08		
rs11552449^a^	*DCLRE1B*					↑ exon 2 inclusion
	Transcript uc001eei	−0.64	8.6E − 14	1.6E − 10	2.4E − 06	
	Transcript uc001eeg	0.26	2.2E − 10	2.3E − 07	4.4E − 05	
	Exon 1:3 junction	−0.48	7.0E − 08	4.5E − 05	4.5E − 03	
	Exon 2	0.24	2.7E − 08	2.1E − 05	6.1E − 06	
rs8170	*BABAM1*					Longer exon 2 (38 bp 5′): ↑ use of 3′ acceptor site at chr19:17 379 565
	Transcript uc002nfv	0.47	3.0E − 08	2.1E − 05	8.2E − 06	
	Transcript uc002nfu	−0.29	2.7E − 07	1.7E − 04	0.038	
rs11249433	*SRGAP2D*					^b^
	Exon 2:3 junction	−0.39	1.3E − 08	1.2E − 05		
rs11552449^a^	*PHTF1*					↑ inclusion of exons 1 and 2
	Exon 1:2 junction	0.47	3.0E − 08	2.1E − 05		
	Exon 2	0.28	1.9E − 06	9.0E − 04		
rs616488	*PEX14*					↓ transcript uc001arm (exons 1, 2, 6, 7)
	Exon 7	−0.39	3.8E − 07	2.2E − 04	0.021	
	Transcript uc001arm	−0.39	5.1E − 07	2.8E − 04		
rs999737	*RAD51L1*					↓ transcript uc001xkf (exons 1–11, exon 14)
	Exon 15	−0.33	2.3E − 05	9.1E − 03		

*β* is for the effect of the breast cancer risk allele on the gene component.

bp, base pairs.

Transcripts are named according to UCSC ID. Genomic positions are for hg19 build.

*P*-values for ER-negative tumors displayed when is <0.05.

^a^SNP is associated with transcript expression of two different genes.

^b^Splicing QTL association not predicted to be linked to breast cancer given pattern of association at locus.

### Relationship between breast cancer risk association and splicing QTL association

Because eQTLs and splicing QTLs are common throughout the genome (9,10,26), it is plausible that a raSNP could be associated with nearby gene or transcript expression but that this association would not be connected to breast cancer. To assess the possibility that the causal SNPs for the splicing QTL and the breast cancer association were different, we calculated the posterior probabilities of each of the SNPs at the locus as the causal SNP for each of the two traits. For the trait of breast cancer risk, we used association statistics from the GAME-ON website (http://gameon.dfci.harvard.edu). We then identified the most parsimonious list of SNPs that produced a cumulative posterior probability of >0.95 for the breast cancer association (Table 2). We then compared the cumulative posterior probability of the splicing QTL associations for the same set of SNPs. We also repeated the inverse analysis, identifying the most parsimonious list of SNPs that produced a cumulative posterior probability of ≥0.95 of the splicing QTL association and found the cumulative posterior probability of the GWAS association from the GAME-ON data (Supplementary Material, Table S9).

**Table 2.** Overlap of the posterior probabilities of the splicing QTL and case–control association statistics based on cumulative probability of case–control association of >0.95

	Target event	Best *P*-value for breast cancer from GAME-ON	Total number of SNPs	Number of SNPs to reach 0.95 probability case–control	Splicing QTL cumulative probability
Locus 1	*STXBP4*	5.2 × 10^−5^	908	54	0.99
Locus 2	*DCLRE1B*	2.0 × 10^−3^	705	337	0.99
Locus 2	*PHTF1*		705	337	>0.99
Locus 3	BABAM1*	5.1 × 10^−3^	588	452	>0.99
Locus 4	*SRGAP2D*	4.0 × 10^−8^	37	3	4.3 × 10^−9^
Locus 5	*PEX14*-exon7	8.3 × 10^−4^	537	90	0.60
Locus 5	*PEX14*-transcript uc001afk		537	90	0.97
Locus 6	*RAD51L1*-exon15	6.9 × 10^−11^	1151	11	9.2 × 10^−8^
Locus 6	*RAD51L1*-transcript uc001xkf		1151	11	0.29

*P-value for *BABAM1* was obtained from the ER-negative analysis from GAME-ON, because this locus was identified by a GWAS for ER-negative breast cancer.

For some of the loci, such as *STXBP4, SRGAP2D* and *RAD51L1*, the breast cancer association is highly significant and thus the number of likely SNPs is relatively small. For the *STXBP4* locus, we were able to narrow the list of SNPs at the locus from 908 to 40 and noted a very high probability (0.99) that the splicing QTL causal variant was also captured in this set. Conversely, starting with the splicing QTL, we narrowed the SNPs down to 23 and found a posterior probability of 0.55 that the GWAS causal variant was also captured in this set. For the *SRGAP2D* locus, starting with the GWAS data, we narrowed the list of likely SNPs from 37 to 3, and the posterior probability that the splicing QTL was determined by these three SNPs was 4 × 10^−7^. Conversely, starting with the splicing QTL data, we only narrowed down the list of SNPs from 37 to 23 but still found that the posterior probability of the causal SNP for GWAS was ∼0.01. Thus, we excluded the possibility that the splicing QTL and breast cancer association are caused by the same variant at this locus. For *RAD51L1*, we narrowed the list of likely SNPs at the locus from 1152 to 11, and the posterior probability of the 11 SNPs for the splicing QTL was 3 × 10^−9^. Similarly, starting with the splicing QTL, we narrowed the signal to five SNPs which had a posterior probability of the GWAS SNP of ∼1 × 10^−9^. Therefore, we also excluded the possibility that the case–control and exon 15 splicing QTL effect are caused by the same variant. Interestingly, when examining the association of each individual exon of the gene with the raSNP (Supplementary Material, Fig. S2), we noted that the raSNP for *RAD51L1* was associated with several different exons, though only exon 15 at FDR < 0.05. In the reconstructed transcript test, the raSNP for *RAD51L1* was also associated (*P* = 7.3 × 10^−4^), though not at FDR < 0.05, with transcript uc001xkf, which includes exon 14 rather than exon 15. This transcript had greater overlap of the posterior probability with the breast cancer association. This analysis suggests that it may be possible to use this Bayesian approach to disentangle not only whether the causal SNP for a splicing QTL and for breast cancer risk are the same but more precisely which transcript is in fact associated with breast cancer risk.

At other loci, the breast cancer association statistics were less significant and consequently our ability to narrow down the set of plausible SNPs, and to dissect the splicing QTL association from the breast cancer association, was more limited. We note that at the *PEX14* locus, the overlap with the posterior probability for breast cancer was higher for transcript uc001arm than for exon 7, hinting at the possibility that it is this entire transcript rather than simply exon 7 that is implicated in breast cancer risk. At the *DCLRE1B/PHTF1* locus, we were unable to definitively rule out one of those genes when we examined the overlap with the posterior probability of breast cancer. Similarly, the posterior probability at the *BABAM1* locus included a large number of SNPs that we could not narrow effectively based on the breast cancer association.

### Leveraging splicing QTLs to identify causal SNPs

Once a raSNP is identified through GWAS, a major challenge is discerning which of the many possible SNPs in LD with the initially identified SNP might be causal (2). The link between the raSNP and the expression of a particular splice junction or exon can shed new light on the identity of the causal SNP, as in some cases only one or a few SNPs in LD with the original raSNP may be likely able to affect alternative splicing by virtue of their location within the gene. Examining all known variants from 1000 Genomes in each splicing QTL region, we were able to narrow the list of possible functional SNPs for three of the six splicing QTLs predicted to be associated with breast cancer (Supplementary Material, Table S8).

In two cases, the association of the raSNP with alternative splice site usage allowed us to identify the specific likely causal SNP. At the *STXBP4* locus, rs6504950 was associated with increased usage of one 3′ acceptor site of intron 5 of *STXBP4* and decreased usage of another that was six bases distant (Fig. 2). Of 177 SNPs in LD with rs6504950 at *r^2^* > 0.6, only one (rs11658717) was in intron 5, where it altered an AG 3′ acceptor site to AA; as expected, with the G allele, virtually all transcripts appeared to use the first of the two possible 3′ acceptor sites, whereas with the A allele, virtually all transcripts appeared to use the second. Interestingly, not only was rs11658717 more associated with the exon 5–6 junctions than was rs6504950, it was also modestly more associated with breast cancer risk (Supplementary Material, Table S8) (27).

Similarly, rs8170 was associated with increased usage of one 3′ acceptor site in intron 1 of *BABAM1* and decreased usage of another. Of 34 SNPs in LD with rs8170 at *r^2^* > 0.6, only one was located in the first intron (Fig. 3). This SNP, rs10424178, lies within the predicted branch point sequence (25) for the second 3′ acceptor site and is 5 bases 3′ of the first 3′ acceptor site. Like rs11658717, rs10424178 was more strongly associated than the index raSNP with the transcript expression (Supplementary Material, Table S8); data for its association with breast cancer risk were not available.

To test whether the alteration of the branch point sequence by rs10424178 caused the predicted splicing difference *in vitro*, we cloned the *BABAM1* exon 2 and flanking intronic sequence into two minigene plasmid vectors, each containing an alternate allele of rs10424178, transfected each vector into cells and measured the gel band intensity of the two *BABAM1* transcript components (28). In all six replicates, the minor allele was associated with relatively lower usage of the second of the two 3′ acceptor sites (Fig. 3e): on average, 3.5% of transcripts had the shorter exon 2 with the minor allele compared with 6.4% with the major allele (paired *t*-test, *P* = 0.02). This pattern corresponded to what was seen in the TCGA RNA-seq data of the tumors, validating the functional effect of rs10424178 at the locus.

## Discussion

The majority of disease raSNPs are in noncoding regions of the genome (1), and these noncoding raSNPs are presumed to influence regulation of nearby genes. Here, we show that six breast cancer raSNPs are associated with differential isoform expression of seven nearby genes in breast tumors. At five of these loci, the top splicing QTL SNPs are also in high LD with the top breast cancer associated SNPs. Our results suggest that regulation of alternative splicing is perhaps nearly as important as mechanism in affecting breast cancer susceptibility as regulation of overall gene expression: there are eight eQTLs that have been reported in breast tissue with these same breast cancer risk loci (4,7).

Current pipelines for discerning the functional effects of GWAS raSNPs focus on eQTL analyses and searching for associated potential causal variants within coding, transcriptional start site or enhancer regions using systematic annotation of the genome for these sites (2,4,6–8,29–31). The findings of this study suggest that splicing QTL analyses, as outlined here, should be included to help illuminate the function of raSNPs and that systematic annotation of genomic regions crucial for splicing will be important in interpreting the results from GWAS. Careful examination of putative associations is essential to determine that an apparent splicing QTL effect is not an artifact of mapping error or bias in RNA-sequencing data. Minigene splicing reporter assays can confirm the splicing effects of the predicted causal SNPs *in vitro*, much as luciferase reporter assays can confirm the effects of predicted causal SNPs in enhancer regions.

In addition to uncovering causal SNPs, splicing QTL associations can help clarify which candidate gene affects breast cancer risk. For example, rs8170 is in LD with SNPs, which lead to missense variants in *ANKLE1* (6), but our analyses implicate *BABAM1* as the causal gene at this locus. This result is consistent with the known interaction of BABAM1 with BRCA1, and with the fact that rs8170 modifies the risk of *BRCA1* mutation carriers (32). Furthermore, beyond identifying the candidate gene, the splicing QTL associations implicate a particular exon or domain of that gene as important in breast cancer risk. For example, not simply the *BABAM1* gene but an extra 38 base pairs of its 5′ untranslated region is associated with increased breast cancer risk. Similarly, two fewer amino acids in the sixth exon of the *STXBP4* gene are associated with increased breast cancer risk. Additional experiments examining the effects of the particular transcripts we identified to be associated with breast cancer risk should enhance our understanding of breast cancer susceptibility.

We also developed an approach that compares the posterior probability of the causal SNPs for the expression phenotype with the breast cancer phenotype. Previous studies have also examined the likelihood that the causal SNP for an expression phenotype is the same as that for a disease trait (33). Our method is similar to the approach of Giambartolomei *et al*. (34) that calculates the posterior probability of the same SNP being causal for the gene expression and disease risk locus. However, our method is different in that it assumes that there is both a real gene expression association and disease association at the locus and that the only two possibilities are that they are due to the same causative SNP versus different causative SNPs. In contrast, Giambartolomei *et al.* consider a wider range of possibilities including ones that do not have an expression association or disease association at the locus. As we started with loci that had been validated as GWAS hits for breast cancer and with a stringent FDR for association for gene expression, the priors of no association for either of these were not valid. Our approach was limited by the *P*-values at some of the loci that were available from GAME-ON. In particular, for the loci defined by rs11552449 and rs8170, the GWAS signals in the GAME-ON dataset are in the range of *P*-values of 0.01 to 0.001, which is likely insufficient to draw conclusions about the co-localization of the GWAS signal. As larger datasets with more comprehensive SNP coverage become available, the other loci may produce clearer results.

Our study has several limitations. First, we used breast tumor tissue, rather than normal tissue, to identify putative splicing QTLs among the breast cancer raSNPs. A challenge in eQTL analyses has been using tumor tissue, of which there is much more available expression data, to identify these effects in the face of the acquired somatic genetic and epigenetic changes that occur within tumors. Methods have been developed to adjust eQTL analyses performed in tumor tissues for certain somatic alterations, including copy number and methylation status (4). These factors are well understood to affect overall gene expression, but their effects on alternative splicing patterns are, to date, less well understood and more difficult to quantify. However, it is becoming clear that methylation of splice sites can lead to variation in alternative splicing (35) and that intragenic translocation events can affect exon inclusion or exclusion, for example in small cell lung cancer (36). It remains possible that these or other recurrent somatic changes, such as somatic mutations affecting splicing (14), could affect alternative splicing patterns in ways unmeasured in our analyses. While these somatic changes may blur the mechanism connecting a raSNP to its associated change in alternative splicing, the associations should remain valid: for example, the association between a raSNP and exon exclusion might in fact be dictated by its association with an intragenic translocation event, with methylation of a splice site or as, initially hypothesized, with the splicing event itself. Repeating splicing QTL analyses in normal tissues as they become available, for example with the development of the Genotype-Tissue Expression database (37), will be instructive. However, ultimately, functional validation of the impact of the genetic variant on the alternative splicing pattern in an *in vitro* setting, as we performed with the rs8170-*BABAM1* association, is necessary to confirm the alternative splicing changes observed in any tissue.

A second limitation is that we limited our assembled transcript data to those produced by TCGA, namely using one transcript definition (UCSC) and one method of transcript assembly (RSEM), though different methods are known to produce different results (19). By requiring associations identified through whole-transcript reconstruction to be supported by the focused tests of exons and junctions, we eliminated some of the erroneous associations caused by transcript assembly. Nonetheless, we found transcript reconstruction valuable, as it hinted at situations where whole transcripts, rather than just exons or splice sites, were implicated in breast cancer risk (*RAD51L1*). Third, we have shown that mapping bias has the potential to generate false-positive results. While we removed all identified problematic associations, it remains possible that there are unknown variants in the exons and junctions generating unmeasured bias. Fourth, we note that the fact that a raSNP is associated with transcript expression does not mean its effect on breast cancer risk is mediated through that transcript. In fact, we were able to exclude this possibility at one locus (*SRGAP2D*). While we attempted to use information about association with breast cancer risk at the loci to determine which splicing QTLs were likely to be connected to breast cancer, functional studies are necessary to confirm the link between change in expression pattern and cancer risk.

In summary, we have identified seven novel associations between SNPs discovered by GWAS for breast cancer and alternatively spliced isoforms of genes in *cis*, five of which are consistent with mediating the association between raSNP and breast cancer risk. These splicing QTL associations help identify likely causal SNPs and candidate genes and also implicate specific alternatively spliced variants of those genes that mediate the effect on breast cancer susceptibility. Our results suggest that SNPs affecting alternative splicing may play an important role in breast cancer and possibly other complex genetic traits.

## Methods

### Germline genotypes and imputation

We downloaded the Affymetrix SNP6.0 germline genotypes from TCGA (http://cancergenome.nih.gov; date of download 17 December 2012). To obtain genotypes for the breast cancer raSNPs that were not directly genotyped in TCGA, as well as other regional SNPs for fine-mapping, we phased using Shapeit V2 (38) and imputed to the 1000 Genomes phase 1 V3 reference panel (39) using IMPUTE2 (40). We used the imputed ‘dosage’ values (that is, the means of the distribution of imputed genotypes) in association analyses, which allows for uncertainty about the true genotype to be incorporated into the association test (41). All 75 breast cancer raSNPs were either directly genotyped in TCGA or had an INFO score of ≥0.5.

### Splicing QTL association analyses

All statistical analyses were performed with the R programing language V2.15.3. We divided ER-positive (*N* = 358) and ER-negative (*N* = 109) tumors based on ER-status in the TCGA clinical data. We performed all analyses on ER-positive and ER-negative tumors separately.

We downloaded the Level 3 TCGA RNA-seq data (http://cancergenome.nih.gov; date of download 30 December 2013) listing the RPKM values and raw reads (used for DEXSeq analysis) for each defined exon counting bin, number of reads mapping to each defined exon–exon junction and RSEM expression estimates for each annotated gene and transcript. We rank-normalized the RPKM values within each exon counting bin, replacing each RPKM value with its fractional rank (that is, its position in the ordered array of all values divided by the total number of values) and transforming that rank onto the standard normal distribution (42). For junction analysis, we adjusted the raw reads value for the number of total reads per sample to obtain RPM values and then rank-normalized these values according to the same method. For transcript analysis, we similarly rank-normalized the RSEM values for the reconstructed transcripts.

For each linear regression analysis (exon, junction and transcript), we adjusted for the overall expression of the gene, genetic ancestry using the first three principal components identified using EIGENSTRAT (43) on the genotypes of all TCGA samples and global expression variability using the first three factors identified using PEER analysis (16,17) on the log2 (RPKM + 0.25) values of exons from the entire TCGA RNA-seq dataset (on ER-positive and ER-negative tumors separately). For both principal component analysis and PEER factor analysis, the first identified principal component or factor explained the vast majority of the variance of the samples, and we chose to use the first three as covariates as there was a subsequent leveling off in proportion of variance explained (Supplementary Material, Fig. S3).

For exon analysis, we tested the association of each raSNP with each exon of each gene containing two or more exons within ±500 kB:

$$\text{RPK}{\text{M}}_{\mathrm{exoni}}=\beta_{0}+\beta_{1}g+\beta_{2}\left[\left(\overset{n}{\underset{\;j=1}{\sum }}\text{RPK}{\text{M}}_{\mathrm{exonj}}\right)-\text{RPK}{\text{M}}_{\mathrm{exoni}}\right]+\beta_{3}PC1+\beta_{4}PC2+\beta_{5}PC3+\beta_{6}K1+\beta_{7}K2+\beta_{8}K3,$$

where $\text{RPK}{\text{M}}_{\mathrm{exoni}}$ is the rank-normalized RPKM for the tested exon, *g* is the genotype at the raSNP (the dosage value where imputed, ranging from 0 to 2), *n* is the number of exons in the gene, PC1 through PC3 are the first three principal components and K1 through K3 are the first three factors identified through PEER (16,17). In the exon analysis, when we adjusted for overall gene expression, we subtracted the RPKM of the tested exon so as not to diminish the power of the test in genes with very few exons, where the overall gene expression may be strongly correlated with the expression of the tested exon. For junction analysis, we tested the association of each raSNP with each exon–exon junction within ±500 kB:

$$\text{RP}{\text{M}}_{\mathrm{junction}}=\beta_{0}+\beta_{1}g+\beta_{2}\text{RSE}{\text{M}}_{\mathrm{gene}}+\beta_{3}PC1+\beta_{4}PC2+\beta_{5}PC3+\beta_{6}K1+\beta_{7}K2+\beta_{8}K3.$$

For transcript analysis, we tested the association of each raSNP with each transcript of each gene with two or more annotated transcripts within ±500 kB, excluding transcripts that had zero expression in >25% of samples:

$$\text{RSE}{\text{M}}_{\mathrm{transcript}}=\beta_{0}+\beta_{1}g+\beta_{2}\text{RSE}{\text{M}}_{\mathrm{gene}}+\beta_{3}PC1+\beta_{4}PC2+\beta_{5}PC3+\beta_{6}K1+\beta_{7}K2+\beta_{8}K3.$$

We used the DEXSeq R Bioconductor software package V1.8.0 (18) to test for differential exon expression between genotypes using raw exon counts and the negative binomial distribution. For dispersion estimates in association tests, given the number of samples, we did not apply exon sharing with fitDispersionFunction(), instead using the dispersion parameter of each exon calculated independently. We used the first ten principal components from EIGENSTRAT as covariates. Supplementary Material, Figure S2 was obtained using the plotDEXSeq() function of the DEXSeq package.

### Evaluation for mapping bias

To assess for evidence of mapping bias, we downloaded the Level 1 RNA-seq BAM files for the 358 ER-positive tumors with matched germline genotypes from TCGA (date of download 7 May 2014). We identified all SNPs from 1000 Genomes phase 1 V3 (39) or HapMap phase 2 (44) that were in LD with the index SNP with *r^2^* > 0.1 in the European (1000 Genomes) or CEU (HapMap) populations and lay within a site (exon or junction) found to be associated with risk genotype. For each relevant association, we counted all reads mapping to the site of interest excluding reads that mapped to those SNPs and recalculated the association with the raSNP genotype, adjusting for overall expression of the gene and genetic ancestry.

### Splicing QTL fine-mapping

For the 358 ER-positive tumors, we obtained the genotypes of all SNPs from 1000 Genomes phase 1 V3 that were within ±500 kB of each index raSNP that had been identified as a splicing QTL, that had minor allele frequency of >0.001 in TCGA samples and, if imputed as described previously, that had an INFO score of >0.5. We then calculated the *P*-value for the association of each of these SNPs with the exon, junction or transcript expression that we had identified as being most significantly associated with the index raSNP. We used the LocusZoom software (23) to generate plots of the splicing QTL *P*-values against genomic position, colored by LD *r^2^* as calculated from the European population in 1000 Genomes. We used Human Splicing Finder V2.4.1 (25) to annotate alternative splice sites and branch point sequences. We searched for *P*-values for breast cancer risk association of SNPs in the GAME-ON meta-analysis of breast cancer risk case–control studies (27); if data for the SNP of interest were not available, we used the SNP with the highest *r^2^* with the index SNP in the European population in 1000 Genomes. Figures 2c and 3c and c were obtained using the UCSC Genome Browser (24).

### Evaluating overlap of GWAS and splicing QTL signal

We first calculated the posterior probabilities that each SNP at the locus was the causal SNP for case–control association. We then repeated this process for the splicing QTL signal. For the case–control association, we downloaded all of the association statistics from the 1MB region around the index GWAS SNP from the GAME-ON website (http://gameon.dfci.harvard.edu). We merged the SNP list between the splicing QTL association analysis, which had been imputed to 1000 Genomes (39) and the case–control association, which had been imputed to Hapmap version II (44), and generated a list of overlap SNPs. To calculate the posterior probabilities for each SNP in the dataset being the causal SNP, we used a modification of the approach we have previously developed (45). We started with the observed vector Z of *z* scores from the case–control association test for each of the SNPs and the observed LD matrix *Σ*, which includes the elements *r*_ij_ for each pair of SNPs where *r* is the LD coefficient where *i* and *j* represent individual SNPs from the list of *n* SNPs at the locus.

For each SNP, *i*, we calculated another matrix *M_i_* whose elements μ*_j_* are equal to *z_i_* × *r_ij_*, which are the expected *z* statistics assuming that SNP *i* is causal (46). We then calculated the likelihood, $\ell_{i}$, of SNP *i* being the causal SNP conditional on the observed vector Z, the expected vector M and the observed matrix, Σ, using the inverse of the multivariate normal distribution: $\ell_{i}=\Phi^{-1}(Z,\;M,\;\Sigma ).$ We repeated this process for each SNP, getting a new vector ℓ of elements $\ell_{i}.$ The posterior probability *P_i_* of each SNP *i* is calculated as follows:

$$P_{i}=\frac{l_{i}}{\sum_{\;j=1}^{n}l_{j}},$$

where *n* is the number of elements in ℓ.

We repeated this analysis for the splicing QTL analysis. To derive *Z* scores for the SNPs from the splicing QTL analysis, we calculated the *Z* statistics based on an inverse normal transformation from *P*-values derived from the linear regression models and used the signs from the β coefficients from the model.

Finally, to determine the overlap between the two sets of signals, we ordered the posterior probabilities from the case–control analysis from highest to lowest. We then identified the minimal number of SNPs required to produce a cumulative posterior probability of >0.95 of having the causal variant. If the cumulative sum of the posterior probability of the GWAS was <0.05 when the cumulative sum of the posterior probability of the splicing QTL association was >0.95, we concluded that the two associations were likely to be due to a different causal variant.

### Minigene splicing reporter assay

We synthesized two sequences corresponding to *BABAM1* exon 2, 50 base pairs of flanking 3′ intronic sequence and 100 base pairs of flanking 5′ intronic sequence, one with the major (C) and one with the minor (T) allele of rs10424178, with an upstream SalI and a downstream XbaI restriction enzyme site (purchased from IDT DNA). We subcloned these sequences into the RHCglo minigene splicing reporter construct (28) by SalI and XbaI restriction enzyme digest and ligation. Both SNP variation subclones were verified by sequencing.

We then plated HEK293T cells at 1 × 10^6^ cells/60-mm culture dish in 4 ml growth medium (DMEM with 10% fetal bovine serum, 1% penicillin–streptomycin and 1% l-glutamine; GIBCO Life Technologies). Twenty-four hours after plating, we transfected cultures with 1 μg of the minigene plasmid using Lipofectamine 2000 (Life Technologies). We extracted total RNA 24 h post-transfection by mirVana miRNA Isolation Kit (Life Technologies). We performed RT–PCR on 1 µg of total extracted RNA to generate cDNA using the iScript cDNA Synthesis Kit (Bio-Rad). We performed PCR on 200 ng of the generated cDNA using the MyTaq Red Mix (Bioline). The upstream primer was RSV5U, and the downstream primer was TNIE4 as previously described (28). PCR products were subject to electrophoresis on a 3% agarose gel and imaged and quantified by ChemiDoc XRS+ (Bio-Rad).

## Supplementary material

Supplementary Material is available at *HMG* online.

## Funding

This work was supported by a Developmental Award from the UCSF Breast Oncology Program and by grants from the NIH K24CA169004, CA120120 to E.Z.; the DoD Era of Hope W81XWH-12-1-0272 and the UCSF Breast Oncology Program Funding from the Atwater Family to A.G.; an NIH R01 GM071655 to S.B.; an NIH T32 Post-doctoral Training Grant (5T32CA108462-10) to A.Z. and a UCSF Graduate Research Mentorship Fellowship to R.C. Funding to pay the Open Access publication charges for this article was provided by grants from NCI (CA169004 and R21CA179442).

## Supplementary Material

Supplementary Data
