# Supplementary material for: Multiple breast cancer risk variants are associated with differential transcript isoform expression in tumors
Source: Hum Mol Genet. 2015 Oct 15;24(25):7421–31. doi: 10.1093/hmg/ddv432 (PMC4664170; doi:10.1093/hmg/ddv432)
Supplement: Supplementary Data [file supp_24_25_7421__index.html]

Multiple breast cancer risk variants are associated with differential transcript isoform expression in tumors — Multiple breast cancer risk variants are associated with differential transcript isoform expression in tumors — Supplementary Data 

# Multiple breast cancer risk variants are associated with differential transcript isoform expression in tumors

## Supplementary Data

Supplementary Data

- Supplementary Data - Docx file
