# Supplementary material for: Multiple breast cancer risk variants are associated with differential transcript isoform expression in tumors
Source: Hum Mol Genet. 2015 Oct 15;24(25):7421–31. doi: 10.1093/hmg/ddv432 (PMC4664170; doi:10.1093/hmg/ddv432)
Supplement: Supplementary Data [file supp_ddv432_ddv432supp.docx]

**Supplementary Table 1:** Breast cancer risk-associated SNPs and tested genes

| **RS #** | **Chr** | **Position (hg19)** | **Genes within +/- 500 kB** | **Reference** |
| --- | --- | --- | --- | --- |
| rs616488 | 1 | 10566215 | *RBP7, UBE4B, KIF1B, PGD, APITD1, CORT, DFFA, PEX14, CASZ1, C1orf127* | (1) |
| rs11552449 | 1 | 114448389 | *MAGI3, PHTF1, RSBN1, PTPN22, BCL2L15, AP4B1, DCLRE1B, HIPK1, OLFML3, SYT6, TRIM33* | (1) |
| rs11249433 | 1 | 121280613 | *FAM72B, HIST2H2BA, FCGR1B, LOC647121* | (2) |
| rs6678914 | 1 | 202187176 | *NAV1, IPO9, SHISA4, LMOD1, TIMM17A, RNPEP, ELF3, GPR37L1, ARL8A, PTPN7, PTPRVP, LGR6, UBE2T, PPP1R12B, SYT2* | (3) |
| rs4245739 | 1 | 204518842 | *SOX13, ETNK2, REN, KISS1, GOLT1A, PLEKHA6, LOC127841, PPP1R15B, PIK3C2B, MDM4, LRRN2, NFASC, CNTN2* | (3) |
| rs12710696 | 2 | 19320803 | *OSR1* | (3) |
| rs4849887 | 2 | 121245122 | *EPB41L5, TMEM185B, RALB, INHBB, LOC84931, GLI2* | (1) |
| rs2016394 | 2 | 172972971 | *DYNC1I2, SLC25A12, HAT1, MAP1D, DLX1, DLX2, ITGA6, PDK1* | (1) |
| rs1550623 | 2 | 174212894 | *RAPGEF4, ZAK, CDCA7* | (1) |
| rs13387042 | 2 | 217905832 | *IGFBP2, IGFBP5, TNP1, DIRC3* | (4) |
| rs16857609 | 2 | 218296508 | *DIRC3, TNS1* | (1) |
| rs6762644 | 3 | 4742276 | *SUMF1, SETMAR, ITPR1, EGOT, BHLHE40, ARL8B, EDEM1* | (1) |
| rs4973768 | 3 | 27416013 | *NEK10, SLC4A7, EOMES* | (5) |
| rs12493607 | 3 | 30682939 | *TGFBR2, GADL1* | (1) |
| rs9790517 | 4 | 106084778 | *TET2, PPA2, EEF1A1P9, ARHGEF38* | (1) |
| rs6828523 | 4 | 175846426 | *HPGD, GLRA3, ADAM29* | (1) |
| rs10069690 | 5 | 1279790 | *ZDHHC11, BRD9, TRIP13, NKD2, SLC12A7, SLC6A19, SLC6A18, TERT, CLPTM1L, SLC6A3, LPCAT1, SDHAP3, LOC728613* | (3) |
| rs4415084 | 5 | 44662515 | *FGF10, MRPS30* | (6) |
| rs889312 | 5 | 56031884 | *MAP3K1, C5orf35, MIER3, GPBP1* | (7) |
| rs10472076 | 5 | 58184061 | *PLK2, GAPT, RAB3C, PDE4D* | (1) |
| rs1353747 | 5 | 58337481 | *RAB3C, PDE4D* | (1) |
| rs1432679 | 5 | 158244083 | *EBF1, RNF145, UBLCP1, IL12B* | (1) |
| rs11242675 | 6 | 1318878 | *LOC285768, FOXQ1, FOXF2, FOXC1, GMDS* | (1) |
| rs204247 | 6 | 13722523 | *PHACTR1, TBC1D7, GFOD1, C6orf114, SIRT5, NOL7, RANBP9, CCDC90A, RNF182, CD83* | (1) |
| rs17530068 | 6 | 82193109 | *FAM46A* | (8) |
| rs2180341 | 6 | 127600630 | *RSPO3, RNF146, ECHDC1, KIAA0408, C6orf174, C6orf58, THEMIS* | (9) |
| rs9485372 | 6 | 149608874 | *UST, TAB2, SUMO4, PPIL4, ZC3H12D, C6orf72, KATNA1, LATS1, NUP43, PCMT1* | (10) |
| rs3757318 | 6 | 151914113 | *MTHFD1L, AKAP12, ZBTB2, RMND1, C6orf211, C6orf97, ESR1* | (11) |
| rs2046210 | 6 | 151948366 | *AKAP12, ZBTB2, RMND1, C6orf211, C6orf97, ESR1, SYNE1* | (12) |
| rs9383951 | 6 | 152295613 | *C6orf97, ESR1, SYNE1* | (10) |
| rs720475 | 7 | 144074929 | *FAM115A, OR2F2, OR2F1, OR6B1, OR2A5, OR2A25, OR2A12, OR2A2, OR2A14, CTAGE4, ARHGEF35, OR2A1, OR2A9P, OR2A7, CTAGE4, OR2A9P, OR2A1, ARHGEF5, NOBOX, TPK1* | (1) |
| rs9693444 | 8 | 29509616 | *KIF13B, DUSP4, C8orf75, LOC286135, TMEM66, LEPROTL1, MBOAT4* | (1) |
| rs6472903 | 8 | 76230301 | *PI15, CRISPLD1, HNF4G* | (1) |
| rs2943559 | 8 | 76417937 | *CRISPLD1, HNF4G* | (1) |
| rs13281615 | 8 | 128355618 | *LOC727677, POU5F1B, MYC, PVT1* | (7) |
| rs11780156 | 8 | 129194641 | *MYC, PVT1* | (1) |
| rs1011970 | 9 | 22062134 | *MTAP, C9orf53, CDKN2A, CDKN2BAS, CDKN2B, DMRTA1* | (11) |
| rs10759243 | 9 | 110306115 | *RAD23B, KLF4* | (1) |
| rs865686 | 9 | 110888478 | *None* | (6) |
| rs2380205 | 10 | 5886734 | *UCN3, TUBAL3, NET1, CALML5, CALML3, ASB13, C10orf18, GDI2, ANKRD16, FBXO18, IL15RA, IL2RA, RBM17, PFKFB3* | (11) |
| rs7072776 | 10 | 22032942 | *C10orf114, C10orf140, MLLT10, DNAJC1* | (1) |
| rs11814448 | 10 | 22315843 | *MLLT10, DNAJC1, BMI1, COMMD3, SPAG6* | (1) |
| rs10822013 | 10 | 64251977 | *ARID5B, RTKN2, ZNF365, ADO, EGR2* | (13) |
| rs10995190 | 10 | 64278682 | *ARID5B, RTKN2, ZNF365, ADO, EGR2* | (11) |
| rs704010 | 10 | 80841148 | *LOC283050, ZMIZ1, PPIF, ZCCHC24, EIF5AL1, SFTPA2* | (11) |
| rs11199914 | 10 | 123093901 | *WDR11, FGFR2, ATE1* | (1) |
| rs2981582 | 10 | 123352317 | *FGFR2, ATE1, NSMCE4A, TACC2* | (7) |
| rs3817198 | 11 | 1909006 | *BRSK2, MOB2, DUSP8, LOC338651, KRTAP5-1, KRTAP5-2, KRTAP5-3, KRTAP5-4, KRTAP5-5, FAM99A, FAM99B, KRTAP5-6, CTSD, SYT8, TNNI2, LSP1, TNNT3, MRPL23, LOC100133545, H19, IGF2, INS-IGF2, IGF2AS, INS, TH, ASCL2, C11orf21, TSPAN32, CD81* | (7) |
| rs3903072 | 11 | 65583066 | *CDC42EP2, DPF2, TIGD3, SLC25A45, FRMD8, NEAT1, MALAT1, SCYL1, LTBP3, SSSCA1, FAM89B, EHBP1L1, KCNK7, MAP3K11, PCNXL3, SIPA1, RELA, KAT5, RNASEH2C, DKFZp761E198, OVOL1, SNX32, CFL1, MUS81, EFEMP2, CTSW, FIBP, CCDC85B, FOSL1, C11orf68, DRAP1, TSGA10IP, SART1, EIF1AD, BANF1, CST6, CATSPER1, GAL3ST3, SF3B2, PACS1, KLC2, RAB1B, CNIH2, YIF1A, TMEM151A, CD248* | (1) |
| rs614367 | 11 | 69328764 | *TPCN2, MYEOV, CCND1, ORAOV1, FGF19, FGF4, FGF3* | (11) |
| rs11820646 | 11 | 129461171 | *ARHGAP32, BARX2, TMEM45B, NFRKB, PRDM10, NCRNA00167, APLP2* | (1) |
| rs7107217 | 11 | 129473690 | *ARHGAP32, BARX2, TMEM45B, NFRKB, PRDM10, NCRNA00167, APLP2* | (10) |
| rs12422552 | 12 | 14413931 | *GRIN2B, ATF7IP, PLBD1, GUCY2C* | (1) |
| rs10771399 | 12 | 28155080 | *C12orf70, PPFIBP1, REP15, MRPS35, LOC100133893, KLHDC5, PTHLH, CCDC91* | (3) |
| rs17356907 | 12 | 96027759 | *FGD6, VEZT, METAP2, USP44, NTN4, SNRPF, CCDC38, AMDHD1, HAL, LTA4H* | (1) |
| rs1292011 | 12 | 115836522 | *None* | (1) |
| rs2236007 | 14 | 37132769 | *MBIP, SFTA3, NKX2-1, NKX2-8, PAX9, SLC25A21* | (1) |
| rs2588809 | 14 | 68660428 | *RDH11, RDH12, ZFYVE26, RAD51L1* | (1) |
| rs999737 | 14 | 69034682 | *RAD51L1, ZFP36L1, C14orf181, ACTN1, DCAF5* | (2) |
| rs941764 | 14 | 91841069 | *RPS6KA5, C14orf159, SNORA11B, GPR68, CCDC88C, SMEK1, C14orf184, CATSPERB, TC2N, FBLN5* | (1) |
| rs3803662 | 16 | 52586341 | *TOX3* | (4) |
| rs4784227 | 16 | 52599188 | *TOX3, CHD9* | (10) |
| rs17817449 | 16 | 53813367 | *CHD9, RBL2, AKTIP, RPGRIP1L, FTO* | (1) |
| rs11075995 | 16 | 53855291 | *CHD9, RBL2, AKTIP, RPGRIP1L, FTO, IRX3* | (3) |
| rs13329835 | 16 | 80650805 | *DYNLRB2, CDYL2, C16orf61, CENPN, ATMIN, C16orf46, GCSH, PKD1L2* | (1) |
| rs6504950 | 17 | 53056471 | *TOM1L1, COX11, STXBP4, HLF, MMD* | (5) |
| rs527616 | 18 | 24337424 | *TAF4B, KCTD1, LOC728606, AQP4, C18orf16, CHST9* | (1) |
| rs1436904 | 18 | 24570667 | *KCTD1, LOC728606, AQP4, C18orf16, CHST9* | (1) |
| rs8170 | 19 | 17389704 | *NWD1, SIN3B, F2RL3, CPAMD8, HAUS8, MYO9B, USE1, OCEL1, NR2F6, USHBP1, C19orf62, ANKLE1, ABHD8, MRPL34, DDA1, ANO8, GTPBP3, PLVAP, BST2, FAM125A, NXNL1, SLC27A1, PGLS, FAM129C, GLT25D1, UNC13A, MAP1S, FCHO1* | (14) |
| rs2363956 | 19 | 17394124 | *NWD1, SIN3B, F2RL3, CPAMD8, HAUS8, MYO9B, USE1, OCEL1, NR2F6, USHBP1, C19orf62, ANKLE1, ABHD8, MRPL34, DDA1, ANO8, GTPBP3, PLVAP, BST2, FAM125A, NXNL1, SLC27A1, PGLS, FAM129C, GLT25D1, UNC13A, MAP1S, FCHO1* | (14) |
| rs4808801 | 19 | 18571141 | *KCNN1, ARRDC2, IL12RB1, MAST3, PIK3R2, IFI30, MPV17L2, RAB3A, PDE4C, KIAA1683, JUND, LSM4, PGPEP1, GDF15, LRRC25, SSBP4, ISYNA1, ELL, FKBP8, C19orf50, UBA52, C19orf60, CRLF1, TMEM59L, KLHL26, CRTC1, COMP, UPF1, GDF1, LASS1, COPE, DDX49, HOMER3* | (1) |
| rs3760982 | 19 | 44286513 | *PRG1, CD177, TEX101, LYPD3, PHLDB3, ETHE1, ZNF575, XRCC1, IRGQ, ZNF576, SRRM5, ZNF428, CADM4, PLAUR, IRGC, C19orf61, KCNN4, LYPD5, ZNF283, ZNF404, ZNF45, ZNF221, ZNF155, ZNF230, ZNF284, ZNF222, ZNF223, ZNF224, ZNF225, ZNF234, ZNF226, ZNF227, ZNF235, ZNF233* | (1) |
| rs2284378 | 20 | 32588095 | *CBFA2T2, NECAB3, C20orf144, C20orf134, E2F1, PXMP4, ZNF341, CHMP4B, RALY, EIF2S2, ASIP, AHCY, ITCH* | (8) |
| rs2823093 | 21 | 16520832 | *NRIP1* | (1) |
| rs6001930 | 22 | 40876234 | *GRAP2, FAM83F, TNRC6B, ADSL, SGSM3, MKL1, MCHR1, SLC25A17, ST13, XPNPEP3, DNAJB7, RBX1* | (1) |

**Supplementary Table 2:** Exons associated with breast cancer raSNPs at FDR < 0.05 in ER-positive tumors. Position is for hg19 build. β is for effect of breast cancer risk allele. FDR is when all exon, junction, and transcript tests are considered together. P-values are shown for ER-negative tumors when < 0.05 and effect size in the same direction. * Association excluded from Table 1 because of possible mapping error/bias.

| **SNP rsID** | **SNP location** | **Gene** | **SNP Distance from Gene** | **β (ER+)** | **P-value (ER+)** | **FDR (ER+)** | **P-value (ER-)** |
| --- | --- | --- | --- | --- | --- | --- | --- |
|  |  | **Exon** |  |  |  |  |  |
| rs720475* | chr7:144,074,929 | *ARHGEF5* | In gene: intron 13 |  |  |  |  |
|  |  | Exon 10 |  | -0.55 | 5.2E-18 | 1.5E-14 | 4.4E-04 |
|  |  | Exon 9 |  | -0.38 | 4.1E-11 | 4.7E-08 |  |
|  |  | Exon 5 |  | 0.17 | 5.9E-07 | 3.1E-04 |  |
|  |  | Exon 8 |  | -0.24 | 6.2E-07 | 3.2E-04 |  |
|  |  | Exon 11 |  | -0.19 | 3.6E-06 | 1.6E-03 |  |
| rs3903072* | chr11:65,583,066 | *MUS81* | 45 kB |  |  |  |  |
|  |  | Exon 5 |  | 0.28 | 3.8E-14 | 8.2E-11 | 7.3E-11 |
|  |  | Exon 6 |  | 0.18 | 7.8E-07 | 3.9E-04 | 2.4E-04 |
| rs720475* | chr7:144,074,929 | *OR2A7* | 100 kB |  |  |  |  |
|  |  | Exon 5 |  | 0.37 | 1.8E-12 | 3.1E-09 | 7.7E-05 |
|  |  | Exon 6 |  | 0.29 | 1.5E-08 | 1.2E-05 |  |
|  |  | Exon 3 |  | -0.21 | 9.4E-06 | 4.1E-03 |  |
|  |  | Exon 2 |  | -0.22 | 1.5E-05 | 6.1E-03 |  |
|  |  | Exon 1 |  | -0.29 | 3.3E-05 | 0.013 |  |
|  |  | Exon 7 |  | 0.16 | 8.9E-05 | 0.032 |  |
| rs4808801* | chr19:18,571,141 | *SSBP4* | 26 kB |  |  |  |  |
|  |  | Exon 3 |  | 0.25 | 2.8E-09 | 2.8E-06 |  |
|  |  | Exon 4 |  | 0.18 | 3.2E-07 | 1.9E-04 |  |
|  |  | Exon 2 |  | 0.21 | 1.1E-06 | 5.1E-04 |  |
| rs11552449 | chr1:114,448,389 | *DCLRE1B* | In gene: exon 1 |  |  |  |  |
|  |  | Exon 2 |  | 0.24 | 2.7E-08 | 2.1E-05 | 6.1E-06 |
| rs616488 | chr1:10,566,215 | *PEX14* | In gene: intron 2 |  |  |  |  |
|  |  | Exon 7 |  | -0.39 | 3.8E-07 | 2.2E-04 | 0.021 |
| rs11552449 | chr1:114,448,389 | *PHTF1* | 146 kB |  |  |  |  |
|  |  | Exon 2 |  | 0.28 | 1.9E-06 | 9.0E-04 |  |
| rs999737 | chr14:69,034,682 | *RAD51L1* | In gene: intron 13 |  |  |  |  |
|  |  | Exon 15 |  | -0.33 | 2.3E-05 | 9.1E-03 |  |
| rs720475* | chr7:144,074,929 | *OR2A9P* | 77 kB |  |  |  |  |
|  |  | Exon 2 |  | 0.22 | 3.0E-05 | 0.012 |  |

**Supplementary Table 3:** Exon-exon junctions associated with breast cancer raSNPs at FDR < 0.05 in ER-positive tumors**.** Position is for hg19 build. β is for effect of breast cancer risk allele. FDR is when all exon, junction, and transcript tests are considered together. P-values are shown for ER-negative tumors when < 0.05 and effect size in the same direction. * Association excluded from Table 1 because of possible mapping error/bias.

| **SNP rsID** | **SNP location** | **Gene** | **SNP Distance from Gene** | **β (ER+)** | **P-value (ER+)** | **FDR (ER+)** | **P-value (ER-)** |
| --- | --- | --- | --- | --- | --- | --- | --- |
|  |  | **Junction** |  |  |  |  |  |
| rs6504950 | chr17:53,056,471 | *STXBP4* | In gene: intron 1 |  |  |  |  |
|  |  | chr17:53076812:+,chr17:53076987:+ |  | -0.73 | 5.5E-24 | 8.3E-20 | 1.9E-11 |
|  |  | chr17:53076812:+,chr17:53076993:+ |  | 0.59 | 1.9E-23 | 1.4E-19 | 1.6E-07 |
| rs3903072* | chr11:65,583,066 | *MUS81* | 45 kB |  |  |  |  |
|  |  | chr11:65629750:+,chr11:65629915:+ |  | 0.63 | 1.0E-22 | 5.1E-19 | 9.6E-13 |
| rs4808801* | chr19:18,571,141 | *SSBP4* | 26 kB |  |  |  |  |
|  |  | chr19:18538233:+,chr19:18538562:+ |  | 0.40 | 1.5E-21 | 5.6E-18 | 2.8E-03 |
| rs720475* | chr7:144,074,929 | *ARHGEF5* | In gene: intron 13 |  |  |  |  |
|  |  | chr7:144070377:+,chr7:144071825:+ |  | -0.53 | 1.1E-14 | 2.8E-11 | 2.0E-03 |
|  |  | chr7:144069521:+,chr7:144069776:+ |  | -0.40 | 4.4E-08 | 3.0E-05 |  |
| rs720475* | chr7:144,074,929 | *ARHGEF34P* (previously *OR2A7*) | 91 kB |  |  |  |  |
|  |  | chr7:143974046:-,chr7:143974301:- |  | 0.58 | 9.9E-12 | 1.5E-08 | 0.015 |
|  |  | chr7:143971996:-,chr7:143973445:- |  | 0.56 | 1.6E-11 | 2.2E-08 | 4.0E-03 |
|  |  | chr7:143973534:-,chr7:143973972:- |  | 0.54 | 3.8E-09 | 3.5E-06 | 7.7E-03 |
| rs11249433 | chr1:121,280,613 | *SRGAP2D* | 151 kB |  |  |  |  |
|  |  | chr1:121116047:+,chr1:121116687:+ |  | -0.39 | 1.3E-08 | 1.2E-05 |  |
| rs11552449 | chr1:114,448,389 | *PHTF1* | 146 kB |  |  |  |  |
|  |  | chr1:114240347:-,chr1:114240884:- |  | 0.47 | 3.0E-08 | 2.1E-05 |  |
| rs11552449 | chr1:114,448,389 | *DCLRE1B* | In gene: exon 1 |  |  |  |  |
|  |  | chr1:114448397:+,chr1:114450631:+ |  | -0.48 | 7.0E-08 | 4.5E-05 | 4.5E-03 |

**Supplementary Table 4:** Reconstructed transcripts associated with breast cancer raSNPs at FDR < 0.05 in ER-positive tumors. Position is for hg19 build. β is for effect of breast cancer risk allele. FDR is when all exon, junction, and transcript tests are considered together. P-values are shown for ER-negative tumors when < 0.05 and effect size in the same direction. * Association excluded from Table 1 because of possible mapping error or because unable to replicate with exon- or junction- tests.

| **SNP rsID** | **SNP location** | **Gene** | **SNP Distance from Gene** | **β (ER+)** | **P-value (ER+)** | **FDR (ER+)** | **P-value (ER-)** |
| --- | --- | --- | --- | --- | --- | --- | --- |
|  |  | **Transcript** |  |  |  |  |  |
| rs11552449 | chr1:114,448,389 | *DCLRE1B* | In gene: intron 13 |  |  |  |  |
|  |  | uc001eei |  | -0.64 | 8.6E-14 | 1.6E-10 | 2.4E-06 |
|  |  | uc001eeg |  | 0.26 | 2.2E-10 | 2.3E-07 | 4.4E-05 |
| rs6504950 | chr17:53,056,471 | *STXBP4* | In gene: intron 1 |  |  |  |  |
|  |  | uc010dcc |  | -0.42 | 3.5E-11 | 4.4E-08 |  |
| rs8170 | chr19:17,389,704 | *BABAM1* | In gene: exon 8 |  |  |  |  |
|  |  | uc002nfv |  | 0.47 | 3.0E-08 | 2.1E-05 | 8.2E-06 |
|  |  | uc002nfu |  | -0.29 | 2.7E-07 | 1.7E-04 | 0.038 |
| rs616488 | chr1:10,566,215 | *PEX14* | In gene: intron 2 |  |  |  |  |
|  |  | uc001arm |  | -0.39 | 5.1E-07 | 2.8E-04 |  |
| rs3817198* | chr11:1,909,006 | *MOB2* | 124 kB |  |  |  |  |
|  |  | uc001ltq |  | 0.34 | 1.0E-05 | 4.3E-03 |  |
| rs720475* | chr7:144,074,929 | *OR2A7* | 100 kB |  |  |  |  |
|  |  | uc011kuc |  | -0.26 | 6.6E-05 | 0.025 |  |

**Supplementary Table 5:** Exons, junctions, and transcripts associated with breast cancer raSNPs at FDR < 0.05 in ER-negative tumors. Position is for hg19 build. β is for effect of breast cancer risk allele. FDR is when all exon, junction, and transcript tests are considered together. * Association excluded from Table 1 because of possible mapping error/bias.

| **SNP rsID** | **SNP location** | **Gene** | **SNP Distance from Gene** | **β (ER-)** | **P-value (ER-)** | **FDR (ER-)** |
| --- | --- | --- | --- | --- | --- | --- |
|  |  | **Component** |  |  |  |  |
| rs6504950 | chr17:53,056,471 | *STXBP4* | In gene: intron 1 |  |  |  |
|  |  | Junction chr17:53076812:+,chr17:53076987:+ |  | -0.85 | 1.9E-11 | 1.4E-07 |
|  |  | Junction chr17:53076812:+,chr17:53076993:+ |  | 0.53 | 1.6E-07 | 6.0E-04 |
| rs3903072* | chr11:65,583,066 | *MUS81* | 45 kB |  |  |  |
|  |  | Exon 5 |  | 0.32 | 7.3E-11 | 3.6E-07 |
|  |  | Junction chr11:65629750:+,chr11:65629915:+ |  | 0.74 | 9.6E-13 | 1.4E-08 |
| rs11552449 | chr1:114,448,389 | *DCLRE1B* | In gene: exon 1 |  |  |  |
|  |  | Transcript uc001eei |  | -0.64 | 2.4E-06 | 7.1E-03 |
|  |  | Exon 2 |  | 0.36 | 6.1E-06 | 0.015 |
| rs8170 | chr19:17,389,704 | *BABAM1* | In gene: exon 8 |  |  |  |
|  |  | Transcript uc002nfv |  | 0.47 | 8.2E-06 | 0.018 |

**Supplementary Table 6:** Splicing QTLs significant at FDR < 0.05 in ER-positive tumors using DEXSeq (15).

| **SNP rsID** | **SNP location (hg19)** | **Gene** | **P-value** | **FDR** |
| --- | --- | --- | --- | --- |
|  |  | **Exon** |  |  |
| rs720475 | chr7:144,074,929 | *ARHGEF5* |  |  |
|  |  | Exon 10 | 5.5E-18 | 4.0E-14 |
|  |  | Exon 9 | 2.9E-11 | 5.3E-08 |
|  |  | Exon 8 | 3.0E-07 | 2.8E-04 |
|  |  | Exon 11 | 6.9E-07 | 5.6E-04 |
| rs720475 | chr7:144,074,929 | *OR2A7* |  |  |
|  |  | Exon 5 | 1.7E-15 | 6.2E-12 |
|  |  | Exon 6 | 2.7E-10 | 3.3E-07 |
|  |  | Exon 3 | 1.6E-06 | 1.2E-03 |
|  |  | Exon 1 | 3.0E-05 | 9.4E-03 |
|  |  | Exon 7 | 6.4E-05 | 1.7E-02 |
| rs3903072 | chr11:65,583,066 | *MUS81* |  |  |
|  |  | Exon 5 | 1.0E-14 | 3.2E-11 |
|  |  | Exon 6 | 1.3E-07 | 1.4E-04 |
| rs616488 | chr1:10,566,215 | *PEX14* |  |  |
|  |  | Exon 7 | 2.2E-06 | 1.5E-03 |
| rs4808801 | chr19:18,571,141 | *SSBP4* |  |  |
|  |  | Exon 3 | 5.3E-06 | 2.8E-03 |
| rs11552449 | chr1:114,448,389 | *PHTF1* |  |  |
|  |  | Exon 2 | 7.1E-06 | 3.5E-03 |
| rs999737 | chr14:69,034,682 | *RAD51L1* |  |  |
|  |  | Exon 15 | 1.3E-04 | 3.5E-02 |

**Supplementary Table 7:** Recalculation of splicing QTL associations excluding reads containing SNPs with *r^2^* > 0.1 with the index SNP. ***** The following genes were not tested using this method because there were no known SNPs with *r^2^* > 0.1 with the index SNP within the exon(s)/junction(s) of interest: *BABAM1, SRGAP2D, PHTF1, PEX14, RAD51L1*

| **Gene*** | **Index SNP rsID** | **Excluded SNP(s) rsID** | ***r^2^*with index SNP** | **Percent reads excluded** | **P-value before exclusion** | **P-value after exclusion** |
| --- | --- | --- | --- | --- | --- | --- |
| **Component** |  |  |  |  |  |  |
| *STXBP4* | rs6504950 |  |  |  |  |  |
| Exon 5-6 junction 1 |  | rs1156287 | 0.86 | 66% | 1.2E-21 | 3.8E-18 |
| Exon 5-6 junction 2 |  | rs1156287 | 0.86 | 77% | 4.1E-23 | 3.3E-14 |
| *MUS81* | rs3903072 |  |  |  |  |  |
| Exon 5-6 junction |  | rs659857, rs545500 | 0.41, 0.41 | 67% | 1.1E-22 | 0.30 |
| Exon 5 |  | rs659857, rs545500 | 0.41, 0.41 | 18% | 2.2E-14 | 0.16 |
| Exon 6 |  | rs659857, rs545500 | 0.41, 0.41 | 40% | 8.1E-07 | 0.20 |
| *SSBP4* | rs4808801 |  |  |  |  |  |
| Exon 2-3 junction |  | No SNPs in exons 2 or 3 |  |  |  |  |
| Exon 3 |  | rs10405636 | 0.90 | 11% | 5.8E-09 | 9.7E-07 |
| Exon 4 |  | rs10405636 | 0.90 | 36% | 3.0E-06 | 0.27 |
| Exon 2 |  | No SNPs in exons 1, 2, or 3 |  |  |  |  |
| *DCLRE1B* | rs11552449 |  |  |  |  |  |
| Exon 1-3 junction |  | rs11552449 | 1.0 | 94% | 1.3E-07 | 2.8E-03 |
| Exon 2 |  | rs11552449, rs3761936 | 1.0, 0.99 | 39% | 3.0E-05 | 2.0E-04 |

**Supplementary Table 8:** Overlap of the posterior probabilities of the splicing QTL and case-control association statistics based on cumulative probability of the splicing QTL association >0.95. * P-value for *BABAM1*was obtained from the ER-negative analysis from GAME-ON, since this locus was identified by a GWAS for ER-negative breast cancer.

|  | Target event | Best p value for breast cancer from GAME-ON | Total # SNPs | # SNPs to reach 0.95 probability case-control | Splicing QTL cumulative probability |
| --- | --- | --- | --- | --- | --- |
|  |  |  |  |  |  |
| Locus 1 | *STXBP4* | 5.2 x 10^-5^ | 908 | 23 | 0.55 |
| Locus 2 | *DCLRE1B* | 2.0 x 10^-3^ | 705 | 2 | 0.038 |
| Locus 2 | *PHTF1* |  | 705 | 28 | 0.16 |
| Locus 3 | *BABAM1** | 5.1 x 10^-3^ | 588 | 3 | 0.072 |
| Locus 4 | *SRGAP2D* | 4.0 x 10^-8^ | 37 | 23 | 0.019 |
| Locus 5 | *PEX14*-exon7 | 8.3 x 10^-4^ | 537 | 29 | 0.42 |
| Locus 5 | *PEX14*-transcript uc001afk |  | 537 | 19 | 0.49 |
| Locus 6 | *RAD51L1*-exon15 | 6.9 x 10^-11^ | 1151 | 5 | 1.4x10^-9^ |
| Locus 6 | *RAD51L1*-transcript uc001xkf |  | 1151 | 21 | 0.087 |

**Supplementary Table 9: Predicted causal SNPs for three splicing QTLs.** Causal SNPs selected based on location relative to alternative splicing event; e.g. for *DCLRE1B* exon 2 expression, rs3761936 is in exon 2 and rs11102701 is in intron 2 (rs11102701 has no linkage disequilibrium information, but is highly associated with the splicing QTL). P-values for breast cancer risk (BrCa) from GAME-ON meta-analysis of 12 breast cancer GWAS (16). * SNP not available in GAME-ON, and no tag SNP available with higher *r^2^* than that between index and causal SNP.

|  | **rs6504950-*STXBP4*** | **rs8170-*BABAM1*** | **rs11552449-*DCLRE1B*** |
| --- | --- | --- | --- |
| **Number of SNPs *r^2^* > 0.6** | 177 | 34 | 12 |
| **Predicted causal SNP(s)** | rs11658717 | rs10424178 | rs3761936, rs11102701 |
| ***r^2^* with index SNP** | 0.88 | 0.92 | 0.99, unknown |
| **P-value for index SNP (BrCa)** | 2.5E-04 | 4.9E-03 | * |
| **P-value for causal SNP (BrCa)** | 9.9E-05 | * | 0.01, * |
| **P-value for index SNP (splicing QTL)** | 5.5E-24 | 3.0E-08 | 8.6E-14 |
| **P-value for causal SNP (splicing QTL)** | 1.3E-25 | 7.7E-09 | 1.4E-13, 8.8E-12 |

**Supplementary Figure 1:**

**
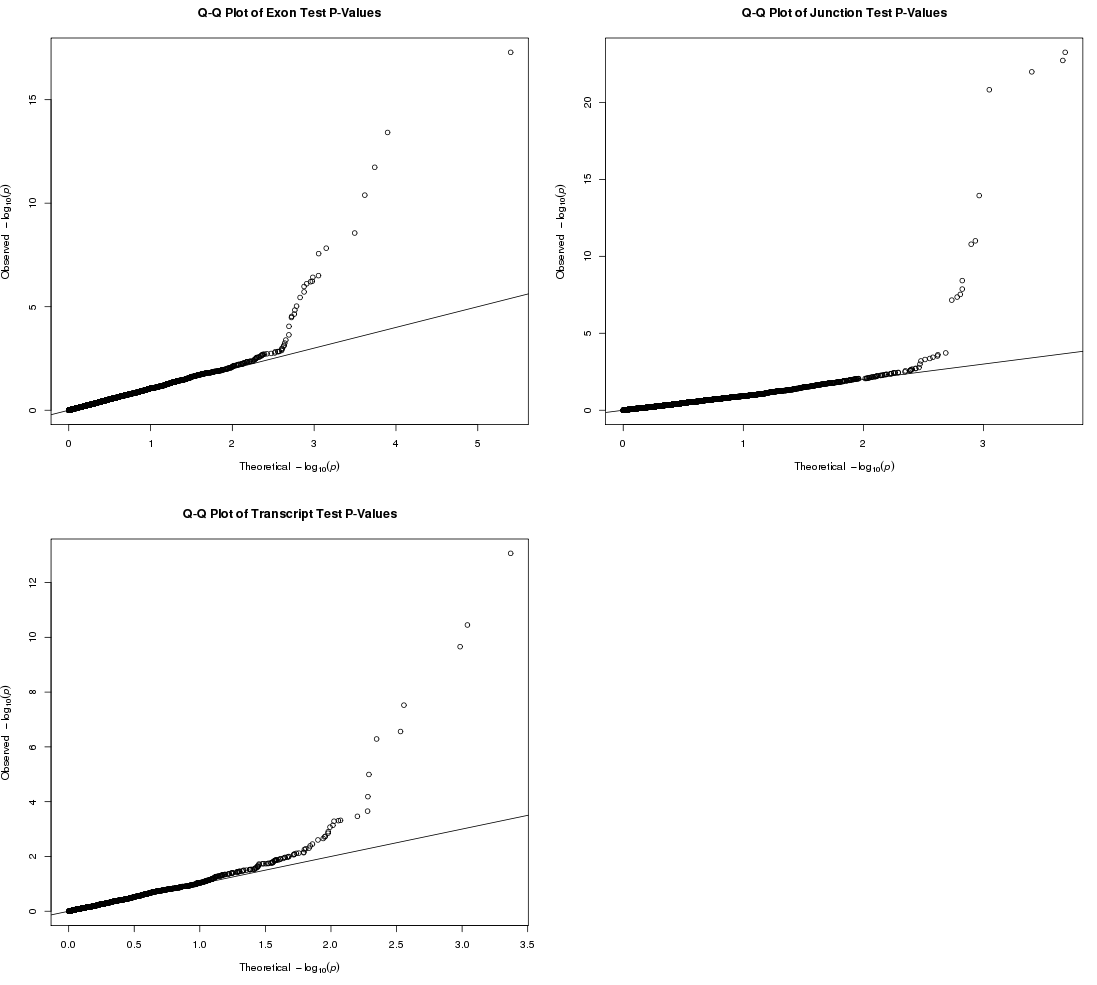
Supplementary Figure 2:**


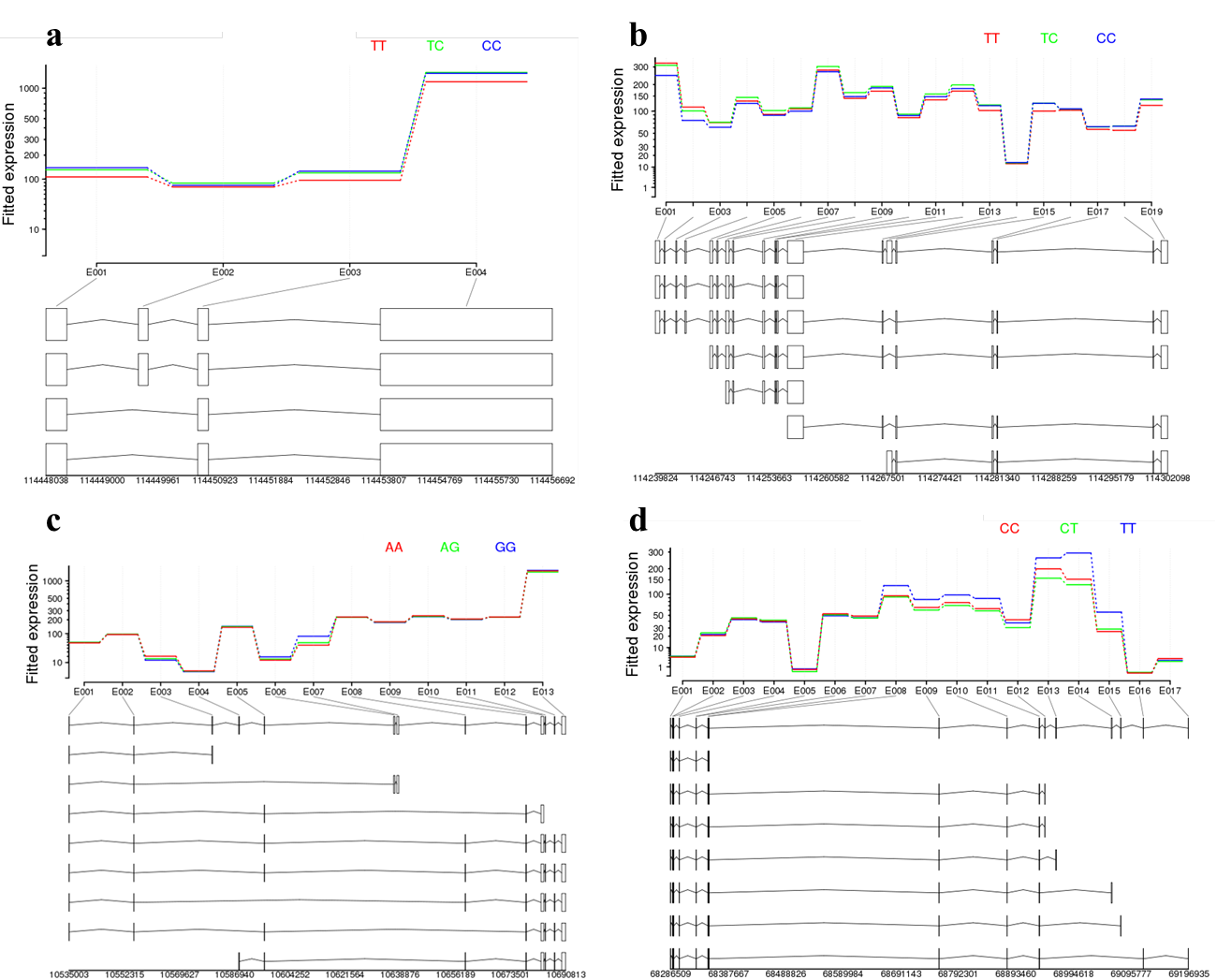


**Supplementary Figure 3:**

**
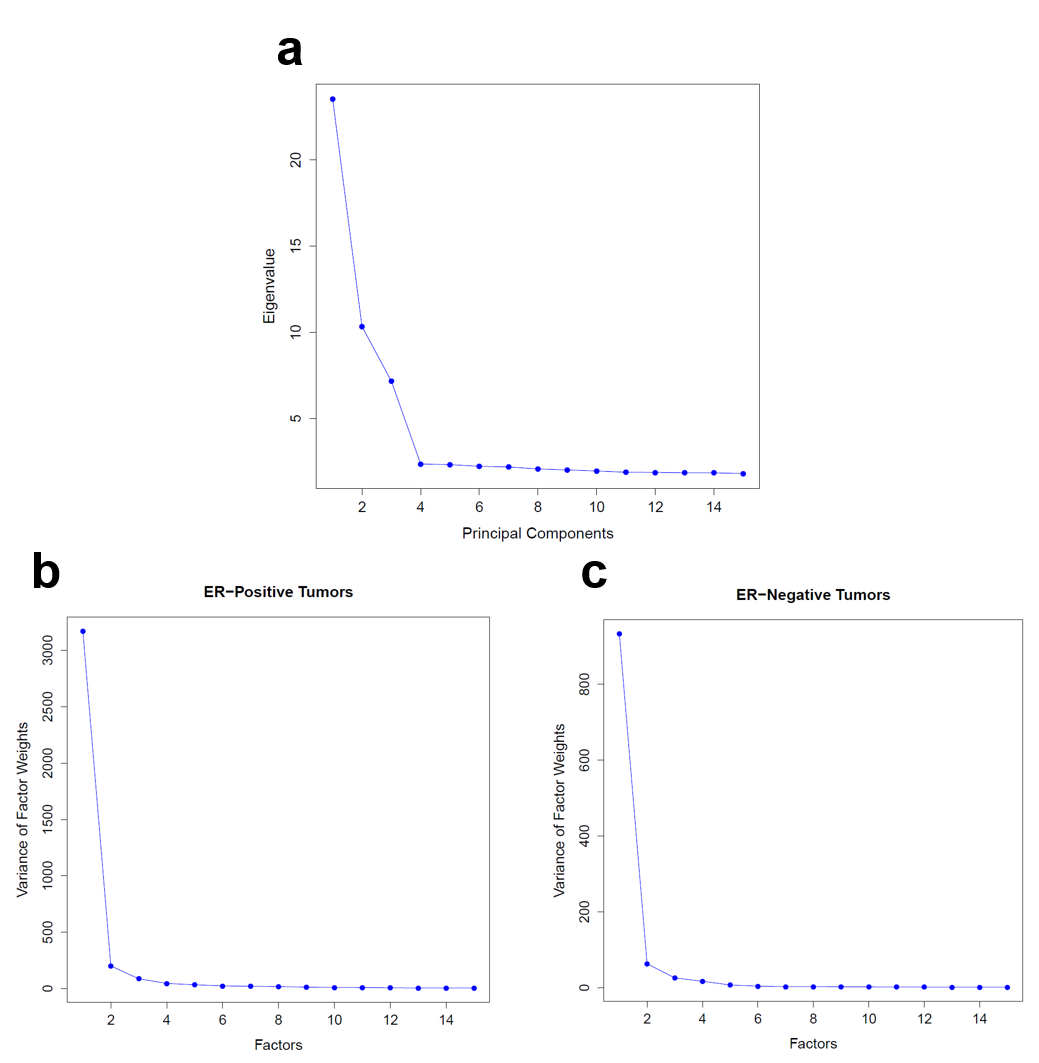
**

**REFERENCES**

1 Michailidou, K., Hall, P., Gonzalez-Neira, A., Ghoussaini, M., Dennis, J., Milne, R.L., Schmidt, M.K., Chang-Claude, J., Bojesen, S.E., Bolla, M.K. *et al.* (2013) Large-scale genotyping identifies 41 new loci associated with breast cancer risk. *Nature genetics*, **45**, 353-361, 361e351-352.

2 Thomas, G., Jacobs, K.B., Kraft, P., Yeager, M., Wacholder, S., Cox, D.G., Hankinson, S.E., Hutchinson, A., Wang, Z., Yu, K. *et al.* (2009) A multistage genome-wide association study in breast cancer identifies two new risk alleles at 1p11.2 and 14q24.1 (RAD51L1). *Nature genetics*, **41**, 579-584.

3 Garcia-Closas, M., Couch, F.J., Lindstrom, S., Michailidou, K., Schmidt, M.K., Brook, M.N., Orr, N., Rhie, S.K., Riboli, E., Feigelson, H.S. *et al.* (2013) Genome-wide association studies identify four ER negative-specific breast cancer risk loci. *Nature genetics*, **45**, 392-398, 398e391-392.

4 Stacey, S.N., Manolescu, A., Sulem, P., Rafnar, T., Gudmundsson, J., Gudjonsson, S.A., Masson, G., Jakobsdottir, M., Thorlacius, S., Helgason, A. *et al.* (2007) Common variants on chromosomes 2q35 and 16q12 confer susceptibility to estrogen receptor-positive breast cancer. *Nature genetics*, **39**, 865-869.

5 Ahmed, S., Thomas, G., Ghoussaini, M., Healey, C.S., Humphreys, M.K., Platte, R., Morrison, J., Maranian, M., Pooley, K.A., Luben, R. *et al.* (2009) Newly discovered breast cancer susceptibility loci on 3p24 and 17q23.2. *Nature genetics*, **41**, 585-590.

6 Fletcher, O., Johnson, N., Orr, N., Hosking, F.J., Gibson, L.J., Walker, K., Zelenika, D., Gut, I., Heath, S., Palles, C. *et al.* (2011) Novel breast cancer susceptibility locus at 9q31.2: results of a genome-wide association study. *Journal of the National Cancer Institute*, **103**, 425-435.

7 Easton, D.F., Pooley, K.A., Dunning, A.M., Pharoah, P.D., Thompson, D., Ballinger, D.G., Struewing, J.P., Morrison, J., Field, H., Luben, R. *et al.* (2007) Genome-wide association study identifies novel breast cancer susceptibility loci. *Nature*, **447**, 1087-1093.

8 Siddiq, A., Couch, F.J., Chen, G.K., Lindstrom, S., Eccles, D., Millikan, R.C., Michailidou, K., Stram, D.O., Beckmann, L., Rhie, S.K. *et al.* (2012) A meta-analysis of genome-wide association studies of breast cancer identifies two novel susceptibility loci at 6q14 and 20q11. *Human molecular genetics*, **21**, 5373-5384.

9 Gold, B., Kirchhoff, T., Stefanov, S., Lautenberger, J., Viale, A., Garber, J., Friedman, E., Narod, S., Olshen, A.B., Gregersen, P. *et al.* (2008) Genome-wide association study provides evidence for a breast cancer risk locus at 6q22.33. *Proceedings of the National Academy of Sciences of the United States of America*, **105**, 4340-4345.

10 Long, J., Cai, Q., Shu, X.O., Qu, S., Li, C., Zheng, Y., Gu, K., Wang, W., Xiang, Y.B., Cheng, J. *et al.* (2010) Identification of a functional genetic variant at 16q12.1 for breast cancer risk: results from the Asia Breast Cancer Consortium. *PLoS genetics*, **6**, e1001002.

11 Turnbull, C., Ahmed, S., Morrison, J., Pernet, D., Renwick, A., Maranian, M., Seal, S., Ghoussaini, M., Hines, S., Healey, C.S. *et al.* (2010) Genome-wide association study identifies five new breast cancer susceptibility loci. *Nature genetics*, **42**, 504-507.

12 Zheng, W., Long, J., Gao, Y.T., Li, C., Zheng, Y., Xiang, Y.B., Wen, W., Levy, S., Deming, S.L., Haines, J.L. *et al.* (2009) Genome-wide association study identifies a new breast cancer susceptibility locus at 6q25.1. *Nature genetics*, **41**, 324-328.

13 Cai, Q., Long, J., Lu, W., Qu, S., Wen, W., Kang, D., Lee, J.Y., Chen, K., Shen, H., Shen, C.Y. *et al.* (2011) Genome-wide association study identifies breast cancer risk variant at 10q21.2: results from the Asia Breast Cancer Consortium. *Human molecular genetics*, **20**, 4991-4999.

14 Antoniou, A.C., Wang, X., Fredericksen, Z.S., McGuffog, L., Tarrell, R., Sinilnikova, O.M., Healey, S., Morrison, J., Kartsonaki, C., Lesnick, T. *et al.* (2010) A locus on 19p13 modifies risk of breast cancer in BRCA1 mutation carriers and is associated with hormone receptor-negative breast cancer in the general population. *Nature genetics*, **42**, 885-892.

15 Anders, S., Reyes, A. and Huber, W. (2012) Detecting differential usage of exons from RNA-seq data. *Genome research*, **22**, 2008-2017.

16 Consortium, G.-O. (2014). Dana-Farber Cancer Institute, Boston, MA, Vol. 2014.

**LEGENDS TO FIGURES**

**Supplementary Figure 1:** Q-Q plots for the exon-, junction-, and transcript-specific tests of association between risk genotype and expression in ER-positive tumors.

**Supplementary Figure 2:** Plotted fitted expression values based on the DEXSeq negative binomial model (15) for each exon by genotype for each splicing QTL identified through exon-specific analysis, after exclusions. Risk genotype is red. Annotated transcripts shown below. (a) rs11552449-*DCLRE1B* (b) rs11552449-*PHTF1* (c) rs616488-*PEX14* (d) rs999737-*RAD51L1*

**Supplementary Figure 3:**

(a) Eigenvalues of the first 15 principal components identified through EIGENSTRAT analysis of the TCGA SNP data. We used the first three principal components as covariates in the linear regression analyses.

(b) Variance of factor weights of the first 15 factors identified through PEER analysis of the ER-positive tumors RNA-seq data (exon RPKM values). We used the first three factors as covariates in the linear regression analyses.

(c) Variance of factor weights of the first 15 factors identified through PEER analysis of the ER-negative tumors RNA-seq data (exon RPKM values). We used the first three factors as covariates in the linear regression analyses.
